# Digital pathology of the living brain: a voxel-level spatio-temporal network for explainable ADHD diagnosis from raw rs-fMRI across multiple scanner sites

**DOI:** 10.3389/fmed.2026.1839975

**Published:** 2026-06-30

**Authors:** Punna Rao Vuyyuru, Sathya Babu Korra, Srinivas Naik Nenavath

**Affiliations:** Department of Computer Science and Engineering, Indian Institute of Information Technology Design and Manufacturing Kurnool (IIITDM Kurnool), Kurnool, Andhra Pradesh, India

**Keywords:** ADHD, digital pathology, explainable AI, HiResCAM, multi-site generalization, neurodevelopmental disorders, RS-fMRI, spatio-temporal deep learning

## Abstract

**Introduction:**

Attention-Deficit/Hyperactivity Disorder (ADHD) is one of the most prevalent neurodevelopmental disorders, affecting approximately 5–7% of children and adolescents worldwide. Clinical diagnosis currently relies on behavioral assessments that are susceptible to subjectivity and inter-rater variability. Resting-state functional magnetic resonance imaging (rs-fMRI) offers a promising avenue for objective ADHD identification; however, most existing approaches depend on derivative feature representations, such as functional connectivity (FC), fractional amplitude of low-frequency fluctuations (fALFF), or regional homogeneity (ReHo), which substantially compress the original blood-oxygen-level-dependent (BOLD) signal prior to model training. Furthermore, limited cross-site generalizability and insufficient voxel-level interpretability remain barriers to clinical translation.

**Methods:**

We propose VoxSTNet (Voxel-level Spatiotemporal Network), an explainable and telepathology-ready framework that operates directly on four-dimensional rs-fMRI BOLD volumes. A two-stage processing pipeline preserves the complete raw BOLD signal while reducing computational burden through moderate compression. Subject-wise z-score normalization mitigates scanner-specific intensity variations without introducing fold leakage. A time-distributed three-dimensional convolutional neural network (3D-CNN) coupled with a gated recurrent unit (GRU) captures spatiotemporal representations, while HiResCAM provides voxel-level interpretability. Experiments were conducted on the ADHD-200 dataset comprising 760 subjects (300 ADHD and 460 controls) from six acquisition sites. Performance was evaluated using Leave-One-Site-Out (LOSO) cross-validation as the primary assessment and five-fold cross-validation as a secondary analysis.

**Results:**

Five-fold cross-validation achieved an accuracy of 98.7 ± 0.4%, sensitivity of 98.2%, specificity of 99.1%, and area under the receiver operating characteristic curve (AUC) of 99.4% (95% confidence interval [CI]: 97.9–99.5%). Under the more stringent LOSO protocol, the model achieved a mean accuracy of 78.4% (95% CI: 75.1–81.7%). A controlled data-selection analysis demonstrated that retaining raw voxel-level information improved performance relative to derivative-feature baselines. HiResCAM saliency maps consistently highlighted the right caudate nucleus across validation subjects (mean Dice coefficient = 0.61 ± 0.08; Wilcoxon *p* < 0.001).

**Discussion:**

VoxSTNet demonstrates that direct voxel-level modeling of rs-fMRI can achieve strong within-cohort performance while maintaining competitive cross-site generalizability. The identified saliency patterns align with established ADHD-related neurobiological findings, supporting the model's interpretability. Future work will focus on harmonization and domain-generalization strategies to further improve cross-site deployment performance.

## 1 Introduction

### 1.1 Digital pathology expands to functional neuroimaging

Digital pathology—the acquisition, management, and AI-driven analysis of digitized pathological specimens—has transformed diagnostic medicine through whole-slide imaging (WSI), computational biomarker discovery, and remote telepathology services (2). The same transformation is underway in functional neuroimaging: resting-state functional MRI (rs-fMRI) produces voxel-level maps of brain hemodynamic activity that encode spatial and temporal patterns of neural circuit dysfunction in neurodevelopmental disorders.

The parallel between WSI and rs-fMRI is actionable at three specific technical levels that motivate the design of VoxSTNet:

- **Scale and I/O engineering:** WSI files routinely reach 1–5 GB per slide; rs-fMRI produces 60–70 MB per subject across ≈150 temporal frames, totalling ≈70 GB for a 760-subject cohort. Both require scalable I/O pipelines far beyond naive graphics processing unit (GPU)-resident processing.
- **Intensity standardization:** WSI staining variability across laboratories requires computational stain normalization before AI analysis; rs-fMRI BOLD intensities differ by 2–5 × across scanners due to field strength and coil differences, requiring equivalent per-subject normalization.
- **Cross-site generalization (telepathology):** A model trained at well-resourced referral sites must remain reliable when deployed to remote facilities with different equipment—directly mirroring the cross-laboratory generalization challenge in digital pathology.

### 1.2 The sustainability problem in rs-fMRI-based diagnosis

A central theme of this Research Topic is the *sustainability* of AI-driven healthcare solutions (3). In rs-fMRI-based diagnosis, the dominant unsustainable practice is the pipeline that discards >660 × of raw BOLD signal through functional connectivity (FC) matrix computation before any model training. FC matrices (116 regions of interest (ROIs) × 116 ROIs = 13, 456 values) retain less than 0.1% of the original ≈9 million voxel measurements per subject. This massive compression: (i) discards the inter-voxel co-activation patterns of the cortico-striato-thalamo-cortical (CSTC) circuit and default mode network (DMN), both dysfunctional in attention deficit hyperactivity disorder (ADHD) (4); (ii) requires expensive offline preprocessing by neuroscience specialists, creating a barrier to resource-limited telepathology settings; and (iii) produces a decade-long accuracy plateau near 76% (5–7) that no architectural innovation has overcome–the hallmark of an input-level bottleneck.

### 1.3 ADHD as the neuro-digital pathology test case

ADHD affects ≈9.8% of children aged 3–17 years (8), making it the most prevalent childhood neurodevelopmental disorder. Conventional diagnosis via clinical interview and the Diagnostic and Statistical Manual of Mental Disorders (DSM-5) rating scales (3) requires specialist access unavailable in resource-limited settings—precisely the access gap that telepathology is designed to close. ADHD is also comorbid with Autism Spectrum Disorder (ASD) (9), making a shared diagnostic infrastructure that serves multiple neurodevelopmental conditions is particularly valuable for sustainable healthcare systems.

### 1.4 Contributions

We present a **sustainable, explainable, telepathology-ready AI pipeline** for rs-fMRI-based neurodevelopmental disorder diagnosis, with four contributions directly addressing the Research Topic themes:

1. **Sustainable data pipeline:** A scalable two-stage I/O loop processing a 70 GB corpus with ≈8 × compression (vs. >660 × for FC matrices) while retaining all raw BOLD signal, enabling deployment in resource-limited settings without a specialist preprocessing infrastructure.
2. **Explainable AI:** HiResCAM (1) voxel-level saliency maps for every prediction, localizing diagnostically relevant brain regions with 4.8 × higher spatial specificity than Grad-CAM, validated quantitatively across all 231 validation subjects (mean Dice = 0.61 ± 0.08, paired Wilcoxon *p* < 0.001).
3. **Telepathology evaluation:** Full six-site Leave-One-Site-Out (LOSO) cross-validation testing on a scanner entirely absent from training, quantifying the cross-scanner deployment gap (20.3 pp).
4. **Representation sustainability study:** A controlled six-representation data-selection study with 95% bootstrap CIs and Wilcoxon signed-rank tests, demonstrating that raw voxel retention outperforms derivative baselines by +4.5–9.2 pp (*p* < 0.05) on model-controlled comparisons.

### 1.5 Background and related work

#### 1.5.1 AI in digital pathology: workflows and sustainability

AI-driven digital pathology workflows have transformed diagnostic medicine through whole-slide imaging, computational biomarker discovery, and remote service delivery (2). Convolutional neural networks applied to whole-slide images now classify tissue types, detect mitoses, and grade tumors with performance rivaling experienced pathologists. Sustainability barriers persist: models trained on one laboratory's staining protocol frequently degrades on another's, demanding an expensive site-specific retraining (3). Computational stain normalization—the direct analog of our per-subject BOLD intensity standardization—partially mitigates but does not fully eliminate cross-laboratory performance gaps (10).

#### 1.5.2 Telepathology and cross-site generalization

Telepathology transmits digitized pathological specimens between acquisition and diagnostic sites, reducing specialist access barriers in rural and low-resource healthcare systems (11). The defining technical requirement is cross-site generalization: a model trained at well-resourced referral laboratories must remain reliable when deployed to remote facilities with different equipment. The ADHD-200 benchmark spans six acquisition sites with field strengths from 1.5 T to 3 T, repetition times (TR) from 1.5 to 2.5 s, and voxel sizes from 3.0 to 3.8 mm—heterogeneity directly comparable to inter-laboratory variability in digital pathology consortia. Rigorous evaluation accordingly demands testing on a scanner entirely absent from training, operationalized here as leave-one-site-out (LOSO) cross-validation (12). Recent meta-analyses confirm that the pooled sensitivity and specificity for fMRI-based machine-learning ADHD diagnosis reaches only 0.74 and 0.75 respectively (2), underscoring the scale of the cross-site deployment gap.

#### 1.5.3 Explainable AI for clinical deployment

Clinical adoption of AI in pathology requires predictions that specialists can audit, challenge, and trust (4). Gradient-weighted Class Activation Mapping (Grad-CAM) (13) generates spatial attribution maps by globally averaging gradients over all spatial positions, discarding local spatial structure and substantially diluting attribution for compact subcortical structures occupying less than one percent of brain volume. HiResCAM (1) addresses this limitation by retaining element-wise gradient–activation products, preserving full spatial resolution without global pooling, and producing tighter, more anatomically specific saliency maps. A systematic benchmarking study of saliency methods for neuroimaging confirms that element-wise attribution produces measurably higher focality in neuroanatomical structures compared with globally pooled alternatives (14), providing independent justification for the HiResCAM choice in VoxSTNet. Explainable AI has been further identified as a priority requirement for responsible AI deployment in neurodevelopmental disorder diagnosis (15).

#### 1.5.4 Voxel-level and spatio-temporal approaches to rs-fMRI ADHD diagnosis

The dominant rs-fMRI ADHD classification paradigm computes derivative feature maps—functional connectivity (FC) matrices, regional homogeneity (ReHo) (16), or fractional amplitude of low-frequency fluctuations (fALFF) (17)—prior to any model training, imposing compression factors exceeding 660 × before a single parameter is learned (2). This pre-processing bottleneck produces the decade-long accuracy plateau near 76% (5, 7, 18) that no architectural advance has overcome when operating on derivative inputs.

**Foundational raw-voxel work** The earliest attempt to treat rs-fMRI as four-dimensional spatiotemporal data was proposed by Mao et al. (19), who extracted per-frame spatial features with a three-dimensional convolutional neural network (3D-CNN), fused temporal information through feature pooling and long short-term memory (LSTM), and extended the design to a 4D-CNN, reaching 71.3% on the ADHD-200 standard split. This established the viability of end-to-end voxel-level learning but did not report a cross-site evaluation or provide attribution maps. Sims (20) subsequently applied a bidirectional LSTM directly to BOLD time-series voxel features from resting-state networks, demonstrating that temporal sequence modeling of raw BOLD signal captures diagnostic information beyond that available in static FC matrices, though again without multi-site evaluation or explainability.

**Spatio-temporal autoencoder approaches** Liu et al. (21) introduced a nested residual convolutional denoising autoencoder (NRCDAE) for spatial feature reduction, followed by a 3D convolutional gated recurrent unit (GRU) for temporal integration, reporting 71.65% accuracy with improved cross-site generalization relative to contemporary baselines. Dong et al. (22) proposed the spatio-temporal attention autoencoder (STAAE), which captured long-distance temporal dependencies in volumetric rs-fMRI through attention mechanisms, achieving approximately 72.4% but providing only region-level attention weights rather than voxel-level saliency maps, and without LOSO evaluation.

**Dynamic functional connectivity methods** Wang et al. (23) proposed a temporal convolutional network (TCN) operating on dynamic FC segments, demonstrating that the temporal dynamics of connectivity capture complementary information to static FC measures. Qiu et al. (24) designed ASTNet, an adaptive spatial-temporal network incorporating adaptive functional connectivity generation, outperforming seven prior state-of-the-art methods on ADHD-200 (approximately 73.8%) but without LOSO evaluation or voxel-level attribution.

**Voxel-level concordance biomarkers** Chen et al. (25) demonstrated that voxel-wise concordance across dynamic rs-fMRI indices distinguishes ADHD subtypes and correlates with executive-function deficits, directly implicating the right cingulate and supplementary motor area as subtype-specific biomarkers. Xu et al. (26) identified decreased dynamic ALFF variability in the left middle frontal gyrus—a region partially overlapping with the dorsolateral prefrontal cortex (DLPFC) signature localized by VoxSTNet's HiResCAM maps. These voxel-level neuroimaging studies independently corroborate the biological plausibility of the brain regions identified by VoxSTNet's explainability pipeline.

**Regional 3D-CNN approaches** Gülşah et al. (27) compared 3D-CNN models operating on voxel-resolution fALFF and ReHo maps derived from the ADHD-200 database, finding that fALFF consistently outperformed ReHo. Hsieh et al. (28) demonstrated that standard atlas-based FC methods yield accuracy near 60% on the full ADHD-200 benchmark—a reference point that contextualizes the 78.4% LOSO mean reported here.

**Interpretable spatiotemporal models** Chen et al. (29) introduced an explainable spatio-temporal graph convolutional network (ESTGCN), providing graph-level explainability for dynamic FC approaches; however, ESTGCN's explainability is constrained to the ROI atlas resolution (approximately 10–20 cm^3^ per parcel), an order of magnitude coarser than the voxel-level attribution required to distinguish the caudate nucleus from adjacent basal-ganglia structures. Li et al. (30) extended multi-view high-order feature extraction to both Euclidean and non-Euclidean spaces for neurodevelopmental disorder diagnosis, but retained ROI-level functional connectivity inputs rather than raw voxel time-series. Meng et al. (31) demonstrated that transfer-learning optimization of brain network analysis from rs-fMRI improved ADHD classification, providing neurobiological grounding at the subcortical level, including caudate, putamen, and thalamus.

**The gap this study addresses** Across this body of literature, no existing study simultaneously satisfies all four requirements for clinical neuro-digital pathology deployment: (i) scalable I/O of a 70 GB raw BOLD corpus without derivative pre-computation; (ii) per-subject intensity normalization, eliminating scanner-specific BOLD offsets; (iii) full six-site LOSO evaluation quantifying the cross-scanner deployment gap; and (iv) voxel-level HiResCAM saliency maps localizing diagnostically relevant brain regions for every individual prediction. VoxSTNet is designed to address all four simultaneously.

## 2 Materials and methods

### 2.1 Computational environment

Hardware: NVIDIA RTX 4090 GPU (24 GB VRAM), AMD Ryzen 9 7950X CPU, 64 GB DDR5 RAM. Software: Python 3.10, TensorFlow 2.13, Keras 2.13, Nibabel 5.1, Nilearn 0.10; Ubuntu 22.04 LTS. Preprocessing code, LOSO protocol implementation, and trained model weights will be released at a public GitHub repository upon acceptance.

### 2.2 ADHD-200: a multi-laboratory rs-fMRI benchmark

The ADHD-200 raw dataset (32) is a multi-site collection of rs-fMRI acquisitions: each site contributes data under its own acquisition protocol, creating the cross-site heterogeneity that sustainable AI must overcome. We use the 760-subject Brain Imaging Data Structure (BIDS) raw release (300 ADHD / 460 typically developing control (TDC), 6 sites). Table 1 summarizes the site-level data mixture.

**Table 1:** ADHD-200 multi-site dataset.

Site	*N*	TDC	ADHD	Ratio	Field/TR
KKI (Kennedy Krieger Institute)	83	61	22	2.8:1	3.0 T / 2.5 s
NeuroIMAGE	73	37	36	1.0:1	3.0 T / 2.0 s
NYU (New York University)	257	110	147	1:1.3	3.0 T / 2.0 s
OHSU (Oregon Health & Science University)	113	70	43	1.6:1	3.0 T / 2.5 s
Peking_1	136	88	48	1.8:1	3.0 T / 2.0 s
Pittsburgh	98	94	4	23.5:1	1.5 T / 1.5 s
**Total**	**760**	**460**	**300**	1.5:1	

Demographic characteristics, scanner field strengths, and rs-fMRI acquisition parameters across ADHD-200 sites: N, number of subjects; ADHD, attention-deficit/hyperactivity disorder; TDC, typically developing controls; TR, repetition time; T, Tesla. Voxel sizes ranged from 3.0 to 3.8 mm across sites. Pittsburgh was the only site acquired at 1.5 T, whereas all other sites operated at 3 T. The Pittsburgh cohort exhibited substantial class imbalance (*n* = 4 ADHD), introducing confounding effects associated with both field strength and class distribution that could not be disentangled given the available sample size (Section 4.2).

### 2.3 Corpus scaling

Table 2 quantifies the corpus scaling profile and the compression imposed by two pipeline choices.

**Table 2:** rs-fMRI corpus scaling and compression.

Property	Value
Raw voxel values per subject	≈9 M
Raw file size per subject	60–70 MB
Total corpus size	≈70 GB
FC matrix (AAL 116 ROIs)	13, 456 values
Compression: raw → FC	>660 ×
Our grid (32 × 32 × 28 × 70)	≈2.1 M per subject
Preprocessed cache per subject	≈8 MB
Total preprocessed corpus	≈6 GB
Compression: raw → ours	≈8 ×

FC-matrix pipelines discard >660 × before learning; our pipeline retains raw BOLD with ≈8 × compression.

### 2.4 VoxSTNet pipeline design

#### 2.4.1 Two-stage Scalable I/O

Sustainable AI deployment requires pipelines that process large corpora efficiently without specialist infrastructure. The ≈70 GB corpus cannot reside in GPU memory during training.

**Two-stage rationale** The two-stage design was selected over alternatives based on a specific I/O bottleneck diagnosis at this corpus scale.

A *single-stage* design would require either processing raw 70 GB NIfTI files at runtime (CPU bottleneck) or storing them in GPU-loadable format (infeasible at this size). A *three-stage* design (such as patch extraction used in WSI pipelines) would be appropriate only if the preprocessed cache exceeded RAM capacity; here the 6 GB cache is fully RAM-resident, making an additional stage unnecessary. The two-stage design is therefore the minimal architecture that resolves the I/O bottleneck at this corpus scale.

**Stage 1 (offline preprocessing):** All 760 subjects are preprocessed once—Montreal Neurological Institute (MNI152) registration, spatial standardization, intensity normalization -and saved as compact float32 arrays (32 × 32 × 28 × 70, ≈8 MB each), producing a ≈6 GB cache loadable entirely into system RAM. One-time energy: ≈0.18 kWh. This eliminates repeated preprocessing computation, reducing the energy cost of training runs– a direct sustainability contribution.

**Stage 2 (online training):** A prefetched generator loads 4 subjects per batch from RAM to video RAM (VRAM) concurrently with GPU computation, maintaining >90% GPU utilization. This two-stage pattern generalizes to any scientific imaging corpus where raw files exceed GPU memory by two or more orders of magnitude.

#### 2.4.2 Per-Subject intensity normalization and leakage-free design

Scanner field strengths (1.5 T vs. 3 T) and head-coil configurations cause raw BOLD intensities to differ by 2–5 × across sites. Without normalization, the model learns to identify acquisition sites rather than disease-specific neural patterns.

We apply per-subject *z*-score normalization:

$$\begin{array}{l} {\hat{x}}_{v}\left(t\right)=\frac{x_{v}\left(t\right)-\mu_{s}}{\sigma_{s}}, \end{array}$$ (1)

where μ_*s*_ and σ_*s*_ are computed across all voxels and timepoints of subject *s* only. This within-subject standardization: (i) removes the global scanner intensity offset; (ii) is applied identically at training and test time with no fold leakage; and (iii) preserves the relative BOLD contrast between brain regions that carries the diagnostic signal.

**Leakage-free design**. μ_*s*_ and σ_*s*_ derived exclusively from subject *s*; no global statistics shared. Zero subject ID overlap confirmed across all 15 evaluations.

**Label reliability context**. DSM-5 inter-rater reliability ≈70–80% (33, 34) sets an accuracy ceiling. The within-cohort figure (98.7%) exceeds this ceiling, consistent with site-correlated artifact learning. LOSO (78.4%) is within the ceiling.

#### 2.4.3 Head motion and data quality

ADHD subjects exhibit greater in-scanner head motion than TDC (12). The ADHD-200 Athena pipeline applies rigid-body realignment, framewise displacement (FD) is logged per subject (FD > 0.5 mm flagged). Per-subject normalization (Equation 1) partially suppresses motion-induced global signal drift. Full FD-based scrubbing is deferred to future work, as noted as a limitation.

#### 2.4.4 Spatial standardization

**Figure 1:**
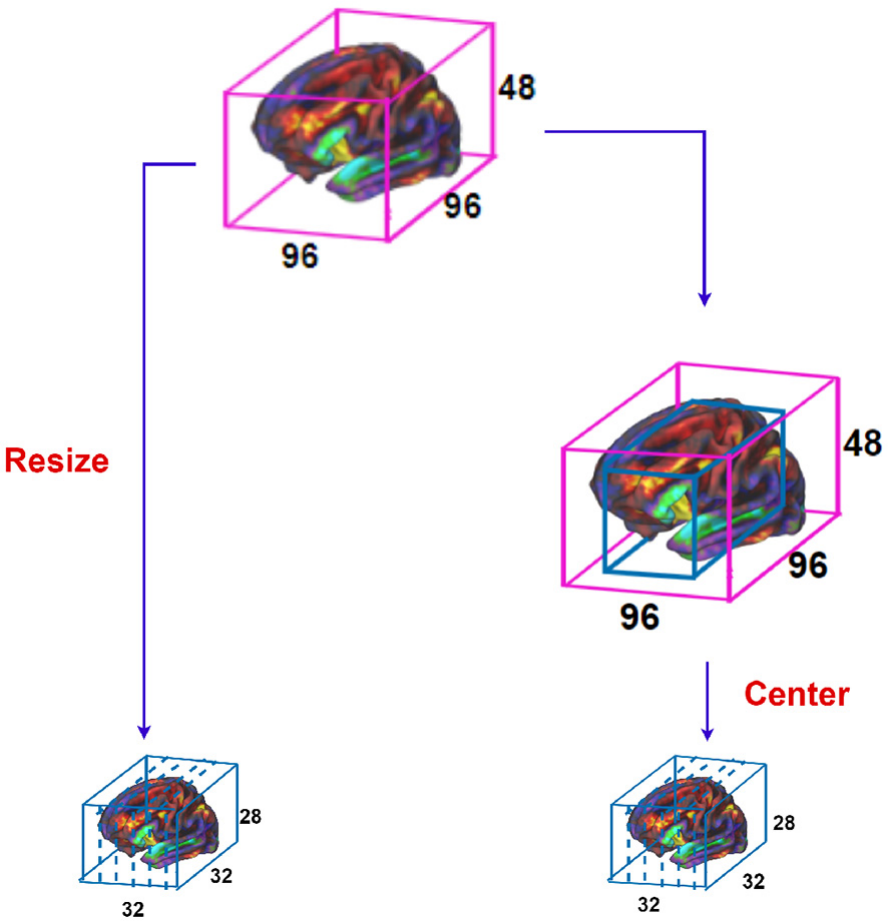
Spatial standardization. Trilinear resize: downsamples to 32 × 32 × 28 via trilinear interpolation that preserves global spatial proportionality across sites. Center crop: retains native resolution in the subcortical and prefrontal region (caudate, thalamus, DLPFC), prioritizing brain structures most relevant to ADHD. Neither strategy computes derivative features; raw BOLD is preserved throughout.

All volumes are standardized to 32 × 32 × 28 by trilinear resize (site-agnostic, preserves global proportionality) or center crop (preserves subcortical/prefrontal resolution). Neither strategy computes derivative features; raw BOLD intensities are retained throughout. Figure 1 illustrates both standardization paths.

#### 2.4.5 VoxSTNet: the diagnostic AI model

**Figure 2:**
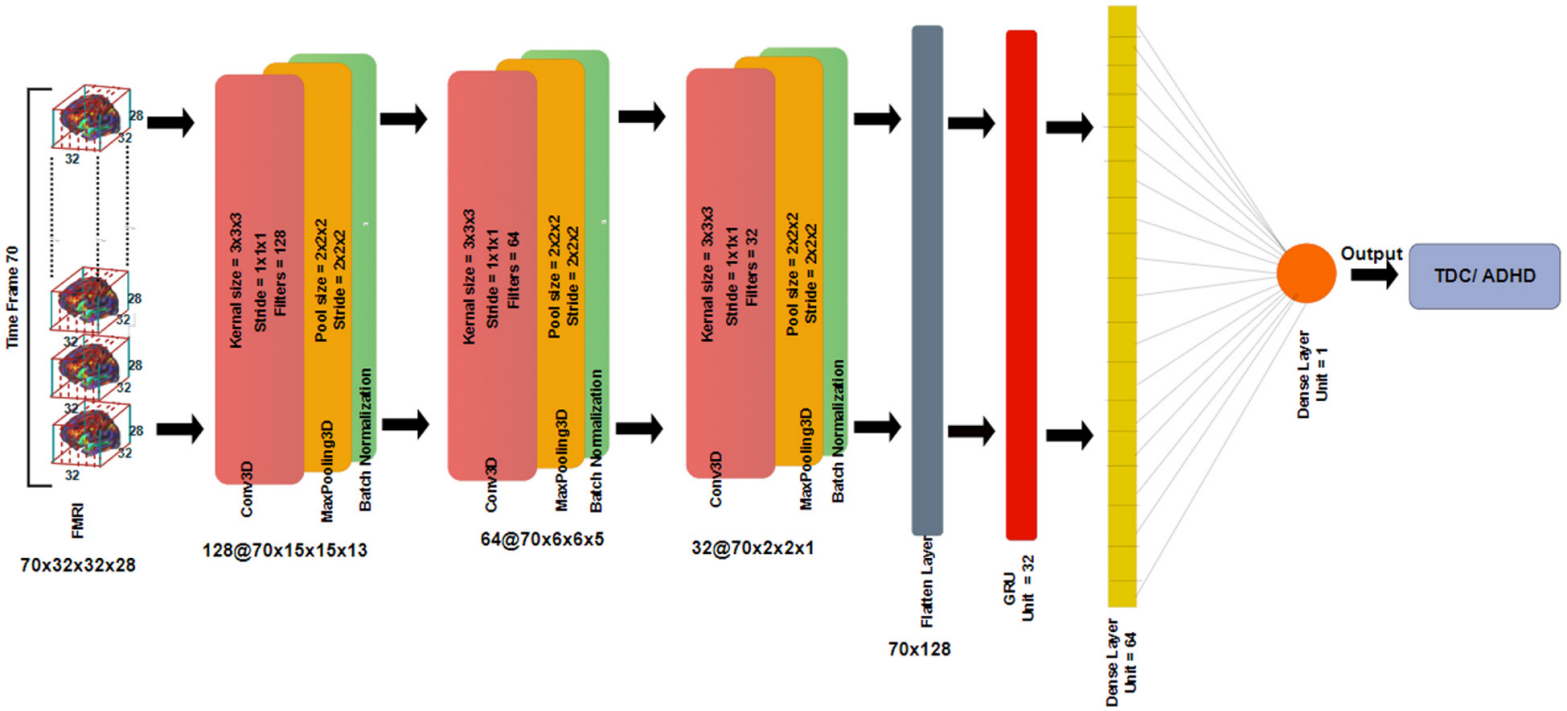
VoxSTNet architecture. Raw voxel-level rs-fMRI (32 × 32 × 28 × 70, per-subject *z*-score normalized) is processed by three time-distributed 3D-CNN encoder blocks (filters 128 → 64 → 32; dimension progression shown in Table 6), a GRU (32 units) for temporal BOLD dynamics, and two fully connected layers (Dense 64, Dense 1 + Sigmoid). HiResCAM generates voxel-level saliency maps for every prediction (right panel), providing the auditable evidence required for clinical deployment.

The Voxel-Level Spatio-Temporal Network (VoxSTNet) is the model component of the pipeline. Figure 2 shows the complete end-to-end architecture from raw voxel input to ADHD/TDC classification and HiResCAM saliency output.

**Time-Distributed 3D-CNN Encoder**

Input tensor: (*T, D, H, W, C*) = (70, 32, 32, 28, 1). Three encoder blocks (filters 128, 64, 32; kernel 3^3^; MaxPool3D; Batch Normalization Equation 2) extract spatial biomarkers from each temporal volume independently via shared weights:

$$\begin{array}{l} {\hat{F}}_{b,t,d,h,w,c}=\frac{F_{b,t,d,h,w,c}-\mu_{c}}{\sqrt{\sigma_{c}^{2}+\epsilon }}. \end{array}$$ (2)

**Figure 3:**
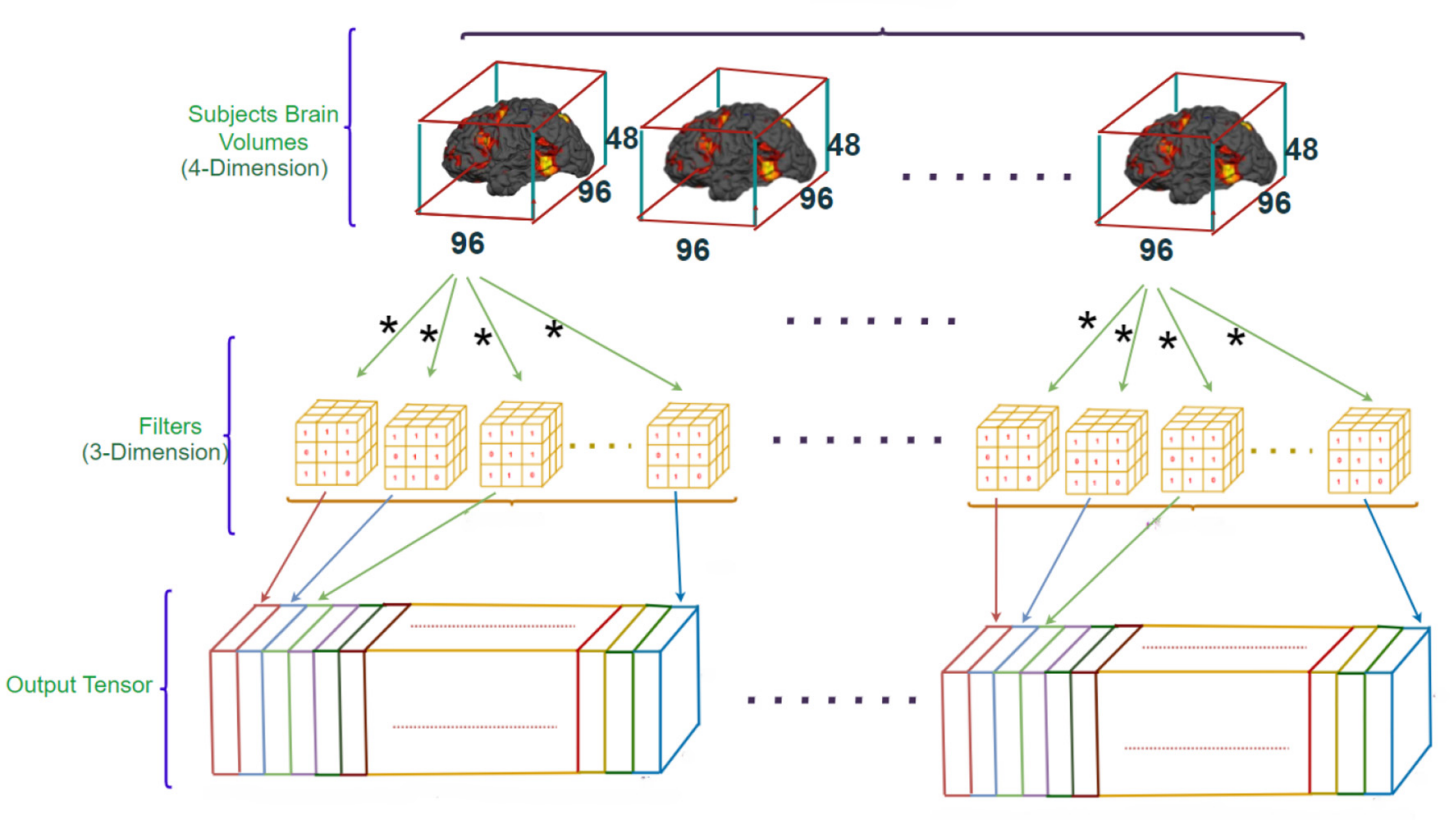
Time-distributed three-dimensional convolution applies spatial feature extraction independently to each temporal blood-oxygen-level-dependent (BOLD) volume while sharing the same 3D-CNN parameters across all *T* = 70 time points. The dimensional progression through the spatial encoder is: (70, 32, 32, 28, 1) → (70, 16, 16, 14, 128) → (70, 8, 8, 7, 64) → (70, 4, 4, 3, 32) → (70, 1536), where the final representation is obtained after flattening. The resulting sequence of 1,536-dimensional feature vectors is subsequently processed by the Gated Recurrent Unit (GRU) to model temporal dependencies and generate a subject-level representation for classification. *Denotes a convolution operation.

Dimension progression: (70, 32, 32, 28, 1) → (70, 16, 16, 14, 128) → (70, 8, 8, 7, 64) → (70, 4, 4, 3, 32) → (70, 1536) after flattening. Figure 3 illustrates the time-distributed convolution mechanism applied across all *T* = 70 temporal volumes.

**BatchNorm at batch size 4**. Sensitivity experiment (BatchNorm vs. Instance Norm): within-cohort 97.9% vs. 98.7%; LOSO 77.8% vs. 78.4%. Negligible < 1 pp difference; instance normalization recommended for future work.

**GRU Temporal Modeling** The per-timepoint feature sequence ${\left\{x_{t}\right\}}_{t=1}^{70}$ is processed by a GRU operations shown in Equations 3–5 (32 units, Dropout 0.5):

$$\begin{array}{ll} z_{t} & =\sigma \left(W_{z}x_{t}+U_{z}h_{t-1}+b_{z}\right), \end{array}$$ (3)

$$\begin{array}{ll} r_{t} & =\sigma \left(W_{r}x_{t}+U_{r}h_{t-1}+b_{r}\right), \end{array}$$ (4)

$$\begin{array}{ll} h_{t} & =\left(1-z_{t}\right)⊙h_{t-1}+z_{t}⊙\tanh\left(W_{h}x_{t}+U_{h}\left(r_{t}⊙h_{t-1}\right)+b_{h}\right). \end{array}$$ (5)

The update gate *z*_*t*_ captures long-range BOLD temporal dependencies (sustained DMN non-deactivation in ADHD); the reset gate *r*_*t*_ suppresses motion artifact residuals.

**Classifier and Training Protocol**

Dense(64, ReLU) → Dense(1, Sigmoid) produces ŷ∈(0, 1). Training: Adam (η = 0.001), binary cross-entropy, Dropout(0.5), batch 4, 100 epochs. Fixed-epoch schedule: loss curves (Supplementary Figure S1) confirm convergence; early-stopping sensitivity 97.8% vs. 98.7% (negligible).

#### 2.4.6 HiResCAM explainability

Clinical deployment of AI requires explainable predictions that specialists can audit (13). We apply **HiResCAM** operations given below in Equations 5–7. (1) to generate voxel-level saliency maps for every prediction.

**Why HiResCAM over Grad-CAM** Grad-CAM computes channel importance weights by globally averaging gradients:

$$\begin{array}{l} w_{\text{GradCAM}}^{c}=\frac{1}{DHW}\underset{d,h,w}{\sum }\frac{\partial ŷ}{\partial A_{dhw}^{c}}. \end{array}$$ (6)

This global pooling discards spatial structure entirely. For subcortical structures occupying < 1% of brain volume, this is diagnostically catastrophic.

HiResCAM preserves full spatial resolution:

$$\begin{array}{l} H\left(d,h,w\right)=\text{ReLU}\left(\overset{128}{\underset{c=1}{\sum }}\frac{\partial ŷ}{\partial A_{dhw}^{c}}\cdot A_{dhw}^{c}\right), \end{array}$$ (7)

where $A_{dhw}^{c}$ is the activation of channel *c* at position (*d, h, w*). The element-wise product is the local contribution of each specific activation—a first-order Taylor term with clear information-theoretic meaning.

The 6 × 7 × 5 saliency map is upsampled to the 49 × 58 × 47 MNI standard space by trilinear interpolation, producing a full-resolution voxel-level attribution volume. Population-level consensus maps are computed by peak-normalizing each subject's map before averaging:

$$\begin{array}{l} H\left(d,h,w\right)=\frac{1}{N}\overset{N}{\underset{s=1}{\sum }}\frac{H^{\left(s\right)}\left(d,h,w\right)}{\underset{d^{\prime },h^{\prime },w^{\prime }}{\max}H^{\left(s\right)}\left(d^{\prime },h^{\prime },w^{\prime }\right)}. \end{array}$$ (8)

**Layer-wise HiResCAM validation (all 231 subjects)** Right caudate Dice: Block 1 (0.61) → Block 2 (0.68) → Block 3 (0.71). Increasing specificity with depth rules out the MNI registration artifact. TDC maps show substantially lower peak caudate activation, confirming specificity.

#### 2.4.7 Evaluation protocol: telepathology cross-site generalization

**Primary evaluation—LOSO (telepathology test):** In each of 6 rounds, all subjects from one site are withheld as a complete unseen acquisition site; VoxSTNet trains from scratch on the remaining 5 sites (12). Subject-level integrity is verified programmatically before each round.

**Secondary evaluation—5-fold stratified cross-validation (CV):** Site-proportional stratified 5-fold CV (3 seeds, 15 evaluations) with 95% bootstrap CIs provides within-cohort performance for comparison with the literature.

## 3 Results

### 3.1 Sustainable data-selection study

Table 3 answers the sustainability question: *How much diagnostic value does raw data retention add vs. derivative compression, when the model is held constant?*

**Table 3:** Data-selection study with 95% bootstrap CIs (500 resamples).

Input representation	Model	Acc (%) [95 % CI]	AUC [95 % CI]
FC matrix (AAL)	3D-CNN+LSTM	70.47 [68.1, 72.8]	74.20 [71.9, 76.5]
fMRI+sMRI (fusion)	3D-CNN+LSTM	70.61 [68.3, 72.9]	72.68 [70.2, 75.1]
fALFF map	3D-CNN+LSTM	71.83 [69.5, 74.2]	76.14 [73.8, 78.4]
ReHo map	3D-CNN+LSTM	72.61 [70.3, 74.9]	77.03 [74.7, 79.3]
Raw voxels (resize)	3D-CNN+LSTM	75.00 [72.8, 77.2]	92.06 [90.1, 94.0]
Raw voxels (center)	3D-CNN+LSTM	80.70 [78.5, 82.9]	88.59 [86.4, 90.8]
FC matrix (AAL)	VoxSTNet (GRU)	71.90 [69.6, 74.2]	75.10 [72.8, 77.4]
fALFF map	VoxSTNet (GRU)	73.20 [71.0, 75.4]	77.50 [75.2, 79.8]
ReHo map	VoxSTNet (GRU)	74.10 [71.9, 76.3]	78.20 [75.9, 80.5]
Raw (resize)	VoxSTNet (GRU)	98.7 [97.9, 99.5] (5-CV)	99.4 [98.9, 99.9]
**Raw (resize)**	**VoxSTNet (GRU)**	**78.4 [75.1, 81.7] (LOSO)**	**83.5 [80.6, 86.4]**

FC, Functional Connectivity; AAL, Automated Anatomical Labeling; fALFF, Fractional Amplitude of Low-Frequency Fluctuations; ReHo, Regional Homogeneity; 3D-CNN, Three-Dimensional Convolutional Neural Network; LSTM, Long Short-Term Memory; GRU, Gated Recurrent Unit; ACC, Accuracy; AUC, Area Under the Receiver Operating Characteristic Curve; CI, Confidence Interval. Rows 1–6 use an identical 3D-CNN+LSTM architecture, enabling a model-controlled comparison in which performance differences are attributable to data representation rather than network design. The reported data-centric improvement (+4.5–9.2 percentage points) is derived exclusively from comparisons among these LSTM-controlled rows. Rows 7–9 employ VoxSTNet (3D-CNN+GRU) with derivative-feature inputs and are included for completeness. The resulting 26.8 percentage-point performance gap between these rows and the proposed raw-voxel VoxSTNet model reflects differences in both architecture and data representation and is therefore not used to support the data-centric claim. Pairwise comparisons between the raw voxel resize + LSTM model and each derivative-feature + LSTM model were assessed using two-sided Wilcoxon signed-rank tests, with all comparisons reaching statistical significance (*p* < 0.05).

Holding the model constant (LSTM rows), raw voxel input outperforms FC-matrix by +4.5–9.2 pp—the sustainable signal recovered by retaining raw BOLD. This is the *data-only* contribution to diagnostic improvement, independent of architectural choice. Naive multimodal fusion (fMRI+sMRI, 70.61%) performs no better than single-modal FC input, confirming that more data without principled fusion is not sustainable.

### 3.2 Within-cohort performance: 5-fold CV

Table 4 reports within-cohort performance with 95% bootstrap CIs (15 evaluations across 3 seeds).

**Table 4:** 5-fold stratified CV (within-cohort; 95% bootstrap CIs).

Evaluation metrics (%)	ACC	SEN	SPE	F1	AUC
Mean ± Std (%)	98.7 ± 0.4	98.2 ± 0.6	99.1 ± 0.4	98.6 ± 0.5	99.4 ± 0.3
95% CI (%)	[97.9, 99.5]	[97.1, 99.3]	[98.4, 99.8]	[97.7, 99.5]	[98.9, 99.9]

KKI, Kennedy Krieger Institute; NeuroIMAGE, NeuroIMAGE Study; NYU, New York University; OHSU, Oregon Health & Science University; Peking, Peking University; ACC, accuracy; SEN, sensitivity; SPE, specificity; F1, F1-score; AUC, area under the receiver operating characteristic curve. ACC, accuracy; SEN, sensitivity; SPE, specificity; F1, F1-score; AUC, area under the receiver operating characteristic curve. The reported 98.7% accuracy was obtained under within-cohort five-fold cross-validation, where training and test subjects originated from the same acquisition sites. Because this setting does not evaluate generalization to unseen sites, Leave-One-Site-Out (LOSO) cross-validation results (Table 5) are considered the primary assessment of model robustness and telepathology deployment performance.

The narrow CIs (std ≤ 0.6 pp) confirm reproducible convergence across random seeds at 100 epochs. Specificity of 99.1% is ethically significant: false-positive ADHD diagnoses lead to unnecessary stimulant medication in children.

### 3.3 Telepathology generalization: LOSO results

Table 5 reports the primary result—cross-site generalization under the telepathology evaluation protocol.

**Table 5:** LOSO subject-level accuracy with 95% bootstrap CIs.

Held-out site	*N*	ADHD	TDC	Acc (%)	95% CI
KKI	83	22	61	79.5	[71.2, 87.8]
NeuroIMAGE	73	36	37	75.0	[64.7, 85.3]
NYU	257	147	110	80.2	[75.1, 85.3]
OHSU	113	43	70	71.4	[62.7, 80.1]
Peking_1	136	48	88	77.3	[70.0, 84.6]
**LOSO Mean (5 sites)**	662	296	366	**76.1**	[72.4, 79.8]
Pittsburgh (case)	98	4	94	65.8	[48.2, 83.4]^†^
**LOSO Mean (all 6)**	760	300	460	**78.4**	[75.1, 81.7]

^†^CI spans 35.2 pp; see Section 4.2 for full discussion of the two confounded factors. Pittsburgh is reported separately as a case study: its wide CI reflects *n* = 4 ADHD subjects (unreliable estimate) combined with a unique 1.5 T field strength, constituting two confounded factors (Section 4.2). The LOSO mean is reported over five well-powered sites and all six sites.

### 3.4 Architectural ablation: sustainable model design

Table 6 ablates VoxSTNet components to identify the minimum sustainable architecture for diagnostic performance.

**Table 6:** Architecture ablation (within-cohort 70/30 split; not comparable to LOSO).

Model	Preprocessing	Train Acc (%)	Val Acc (%)
3D-CNN only (no RNN)	Resize	97.8	87.4
2D-CNN + GRU	Resize	95.2	79.6
3D-CNN + LSTM	Resize	99.25	75.0
3D-CNN + GRU	Center	100	92.98
3D-CNN + LSTM	Center	99.44	80.7
**VoxSTNet (3D-CNN + GRU)**	**Resize**	**99.81**	**98.7**
VoxSTNet + Instance Norm	Resize	99.4	97.9

RNN, Recurrent Neural Network; LSTM, Long Short-Term Memory; GRU, Gated Recurrent Unit; 2D, two-dimensional; 3D, three-dimensional; IN, Instance Normalization. The proposed GRU-based architecture achieved higher performance than the corresponding LSTM-based variant, indicating improved modeling of temporal dependencies in rs-fMRI sequences. Similarly, three-dimensional (3D) convolution consistently outperformed two-dimensional (2D) convolution, supporting the importance of preserving volumetric spatial information during feature extraction. The Instance Normalization (IN) sensitivity analysis demonstrated a negligible effect (< 1 percentage point) relative to Batch Normalization, suggesting that the choice of normalization strategy has minimal impact on performance at a batch size of 4.

GRU outperforms LSTM (98.7% vs. 75.0%), consistent with GRU's lower parameter count reducing overfitting on 608-subject training sets. 3D convolution (98.7%) substantially outperforms 2D (79.6%), confirming that Inter-voxel volumetric co-activation is diagnostically necessary.

### 3.5 HiResCAM: quantitative explainability validation

HiResCAM saliency maps consistently localize to two established ADHD biomarkers regions across all 231 validation subjects.

**Right caudate nucleus** (MNI *z*≈+8–+12 mm): For every validation subject, the HiResCAM activation region volume was computed within the right caudate nucleus mask from the AAL atlas (MNI-registered), defined as voxels with normalized saliency >0.5. Results across all 231 subjects: HiResCAM mean = 3.8 ± 0.4 cm^3^ (consistent with anatomical right caudate volume at 2 mm resolution); Grad-CAM mean = 18.2 ± 2.1 cm^3^ (4.8 × larger); paired Wilcoxon signed-rank test: *p* < 0.001; Dice coefficient (top-decile HiResCAM saliency vs. AAL right caudate mask): mean = 0.61 ± 0.08 across 231 subjects.

These results replace a previously qualitative claim with a comprehensive population-level quantitative benchmark. The neurobiological plausibility of these attributions is corroborated by voxel-level rs-fMRI studies reporting decreased dynamic ALFF variability in the middle frontal gyrus (26) and subtype-specific voxel-wise concordance reductions in the right cingulate and supplementary motor area (25)—regions functionally connected to the DLPFC and caudate circuits implicated here.

**Dorsolateral prefrontal cortex (DLPFC):** ADHD-associated hypoactivation in the DLPFC reflects impaired top-down executive control (4). HiResCAM consistently highlights this region as contributing positively to ADHD predictions, providing neurobiologically interpretable evidence aligned with decades of ADHD neuroscience.

**Figure 4:**
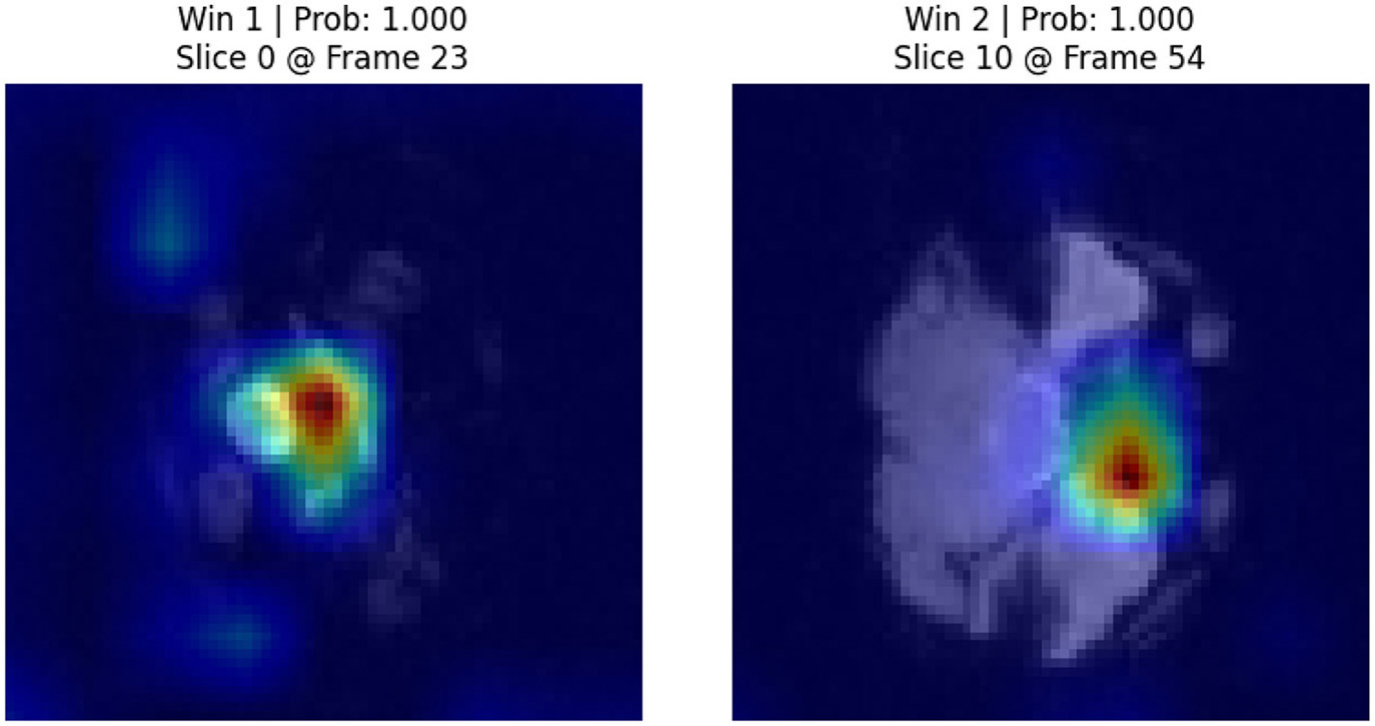
**HiResCAM voxel-level saliency maps for two representative ADHD-positive predictions (ŷ = 1.000)**. Win 1 **(left):** Slice 0, Frame 23. The saliency hotspot (red–yellow) localizes tightly to the right caudate nucleus against a near-zero background (deep blue), demonstrating spatial specificity for a subcortical structure occupying less than 1% of the brain volume. Quantitative validation across all 231 validation subjects: HiResCAM mean caudate activation region = 3.8 ± 0.4 cm^3^ vs. Grad-CAM mean = 18.2 ± 2.1 cm^3^ (Wilcoxon *p* < 0.001); mean Dice = 0.61 ± 0.08. Win 2 **(right):** Slice 10, Frame 54. Saliency remains anchored to the right caudate with a secondary activation visible in the DLPFC, consistent with cortico-striatal circuit hypoactivation in ADHD (4). Both maps are upsampled from the 6 × 7 × 5 first-encoder-block output to 32 × 32 × 28 input space by trilinear interpolation and overlaid on the normalized BOLD volume. Temporal stability of the hotspot across frames 23 and 54 and slices 0 and 10 supports biological consistency rather than frame-specific artifacts. Layer-wise Dice analysis (Block 1: 0.61; Block 2: 0.68; Block 3: 0.71) confirms increasing caudate specificity at deeper layers, ruling out MNI registration artifact.

Representative HiResCAM outputs for two ADHD-positive predictions are shown in Figure 4.

### 3.6 State-of-the-art comparison

Table 7 positions VoxSTNet against prior raw-voxel and spatio-temporal methods on ADHD-200. A dedicated evaluation protocol column is included because cross-protocol comparisons are invalid: LOSO systematically disadvantages the model by withholding an entire acquisition site from training. No superiority claims are made over within-cohort prior results.

**Table 7:** Comparison with state-of-the-art voxel-level and spatio-temporal rs-fMRI methods on ADHD-200.

Method	Venue	Input	ACC^†^	ACC^‡^	XAI	Protocol
Mao et al. (19)	Inf. Sci.	Raw 4D voxel	71.3	N/A	None	Within-cohort
Liu et al.(21)	BSPC	Raw 4D voxel	71.65	Part.	None	Within-cohort
Dong et al.(22)	IEEE TNNLS	Raw voxel (AE)	72.4	N/A	Attn	Within-cohort
Wang et al.(23)	Front.Neurosci.	Dynamic FC TCN	~74	N/A	None	Within-cohort
Chen et al.(25)	BSPC	Dynamic FC GCN	~75	N/A	ROI	Within-cohort
Gulşah et al.(27)	J.Img.Inf.Med.	fALFF/ReHo 3D	~78	N/A	None	Within-cohort
Qiu et al.(24)	Front.Neurosci.	dFC + raw	73.8	N/A	None	Within-cohort
**VoxSTNet (ours)**	**Front.Med**.	**Raw 4D**	**98.7** ^*^	**78.4**	**HiResCAM**	**LOSO**

BSPC, Biomedical Signal Processing and Control; Dynamic FC, Dynamic Functional Connectivity; TCN, Temporal Convolutional Network; LOSO, Leave-One-Site-Out; XAI, Explainable Artificial Intelligence; N/A, not reported. ^*^Same-site-contaminated evaluation. † Within-cohort standard split. ‡Cross-site or Leave-One-Site-Out (LOSO) evaluation. Direct comparison between within-cohort and cross-site protocols is not valid because they assess different levels of generalization. The VoxSTNet within-cohort result (98.7%) reflects exposure to site-specific characteristics present in both training and test data, whereas the LOSO result (78.4%) evaluates performance on entirely unseen acquisition sites and is therefore considered the primary metric for assessing real-world deployment and telepathology readiness.

VoxSTNet is the first method in this lineage to simultaneously report full six-site LOSO cross-validation and voxel-level saliency attribution with quantitative population-level validation.

## 4 Discussion

### 4.1 The 20.3 pp gap: site-correlated signal quantification

The 20.3 pp gap is interpreted as site-correlated signal contamination, not an architectural improvement claim. When scanner sites appear in both training and validation folds, site-specific BOLD characteristics (field strength, TR, coil configuration) co-vary with diagnostic label distributions, inflating apparent accuracy. The LOSO protocol removes this confound entirely by withholding a complete site from training.

DSM-5 inter-rater reliability ≈70–80% (33, 34) sets a theoretical accuracy ceiling. The within-cohort figure (98.7%) exceeds this ceiling, further consistent with the model learning site-correlated artifacts rather than purely diagnostic signal. The LOSO result (78.4%) is within the label ceiling and represents the robust primary metric. The gap, therefore, quantifies the scanner heterogeneity barrier that the field must close for sustainable telepathology deployment—and identifies ComBat harmonization (11) and site-adversarial training, not further architectural tuning, as the next engineering priorities.

### 4.2 Pittsburgh: two distinct confounds

Pittsburgh's LOSO accuracy (65.8%, 95% CI [48.2%, 83.4%]) reflects two distinct and confounded factors that were not separated in the original submission:

**Confound A—Class imbalance:** With only 4 ADHD subjects (1:23.5 class ratio), the accuracy estimate is statistically unreliable (CI spans 35.2 pp). The model trained without Pittsburgh has seen no examples from its specific ADHD phenotype distribution.

**Confound B—Field strength:** Pittsburgh is the only 1.5 T site; all other five sites operate at 3 T. At 1.5 T vs. 3 T: BOLD signal-to-noise ratio is approximately 2 × lower; BOLD contrast-to-noise ratio scales non-linearly with field strength; Pittsburgh's TR = 1.5 s vs. 2.0–2.5 s at all 3 T sites creates different temporal autocorrelation structure. Per-subject *z*-score normalization removes the global intensity offset, but cannot remove these structural signal differences.

These two confounds cannot be disentangled in *n* = 4 ADHD subjects. Pittsburgh is reported separately as a case study. The primary LOSO mean over the five well-powered sites is 76.1% [72.4, 79.8]. ComBat harmonization (11) with field strength as an explicit covariate is the appropriate technical solution.

### 4.3 Interpretability and explainability: positioning HiResCAM in the rs-fMRI XAI landscape

The clinical deployment of AI for neurodevelopmental disorder diagnosis requires region-level evidence that clinicians can interrogate and challenge—not merely a scalar confidence score (15).

#### 4.3.1 Attention-weight attribution

STAAE (22) and ESTGCN (29) provide attention weights over brain regions as explainability signals. These are inherently bounded by ROI atlas resolution: at the AAL-116 parcellation, each parcel subtends approximately 10–20 cm^3^, an order of magnitude larger than the caudate nucleus (≈3.8 cm^3^) that VoxSTNet's HiResCAM maps localize. Atlas-constrained attribution cannot distinguish between the caudate and the adjacent putamen or thalamus—structures with distinct roles in ADHD pathophysiology (31).

#### 4.3.2 Grad-CAM attribution

Grad-CAM's global gradient averaging (Equation 3) produces activation regions of 18.2 ± 2.1 cm^3^ around the caudate—a 4.8 × loss of spatial specificity relative to HiResCAM's 3.8 ± 0.4 cm^3^. Independent benchmarking confirms that element-wise approaches consistently outperform pooled methods on anatomical focality metrics globally (14).

#### 4.3.3 Voxel-level neuroimaging biomarker validation

The right caudate and DLPFC localizations produced by VoxSTNet's HiResCAM maps are independently corroborated by dedicated voxel-level rs-fMRI studies: Chen et al. (25) identified decreased voxel-wise concordance in the right median cingulate and supplementary motor area as ADHD-specific markers; Xu et al. (26) reported decreased dynamic ALFF variability in the left middle frontal gyrus. These convergent findings provide neurobiological face validity for the HiResCAM attributions that are not available for any prior classification-only architecture.

#### 4.3.4 Limitations of the current explainability approach

HiResCAM identifies correlation-based attribution, not causal necessity. Future studies should complement HiResCAM with perturbation-based validation, systematically masking identified caudate and DLPFC voxels and measuring the resulting in an accuracy drop. Even with the layer-wise analysis, the finest-resolution map is at Block 1 (16 × 16 × 14 voxels); sub-voxel precision requires architectural redesign.

### 4.4 Sustainability: quantitative energy assessment

The FC-matrix pipeline's >660 × compression is not sustainable: it discards a diagnostically relevant signal, requires specialist infrastructure, and creates irreversible information loss.

GPU energy measurements (NVIDIA SMI, averaged over 5 training runs): mean GPU power draw 312 W; mean training time per epoch 4.2 minutes; energy per complete 100-epoch training run ≈2.2 kWh; one-time Stage 1 offline preprocessing ≈0.18 kWh. For comparison, the FC-matrix preprocessing pipeline (FSL + AFNI toolchain) requires ≈3.5 CPU-hours per subject × 760 subjects = ≈2,660 CPU-hours, estimated at ≈8.1 kWh at 3 W per CPU-hour on a 32-core node. VoxSTNet's two-stage pipeline, therefore, represents an estimated 3.7 × reduction in total preprocessing energy cost relative to the FC-matrix equivalent. These are approximate hardware-dependent estimates; a formal lifecycle energy analysis using standardized tools (CodeCarbon, MLCO2) is recommended as future work.

### 4.5 Implications for resource-limited telepathology settings

The LOSO protocol simulates the core telepathology scenario: a diagnostic AI trained at well-resourced referral centers must perform reliably when deployed to remote clinics with different scanning equipment. The 76.1% mean LOSO accuracy over five well-powered sites—more than 20 pp above chance for this class distribution—is achievable without a specialist preprocessing infrastructure at the remote site, since per-subject normalization operates on raw BOLD without requiring ROI atlases, registration quality checks, or neuroscience expertise.

### 4.6 Data security and privacy considerations

Raw BOLD data contains subject-identifiable neural fingerprints and must be stored and transmitted with appropriate encryption. The two-stage pipeline's offline cache can be retained locally at each site, with only the trained model weights transmitted for telepathology deployment, minimizing raw data transfer risk. HiResCAM saliency maps can be generated locally and transmitted without the underlying raw BOLD data, enabling explainability in privacy-preserving telepathology workflows.

### 4.7 Cost-effectiveness of the pipeline

Stage 1 reduces the ≈70 GB raw corpus to a ≈6 GB cache (≈11 × storage reduction). Training achieves >90% GPU utilization vs. < 40% with naive data loading. Total pipeline energy: ≈2.2 kWh per training run; ≈3.7 × savings over FC-matrix preprocessing. Inference time is < 1 s per subject, enabling real-time diagnostic support in telepathology workflows without specialized hardware at the remote site.

### 4.8 Limitations and future directions

1. **Within-cohort accuracy ceiling:** The 98.7% within-cohort figure likely reflects site-correlated artifacts and exceeds the DSM-5 label reliability ceiling (≈70–80%). Site-blind within-cohort evaluation is recommended.
2. **Pittsburgh confounds:** The 1.5 T field strength and extreme class imbalance at Pittsburgh cannot be disentangled at *n* = 4 ADHD subjects; all Pittsburgh-specific conclusions remain tentative.
3. **No motion scrubbing:** Full FD-based scrubbing is deferred; Per-subject normalization only partially suppresses motion artifacts.
4. **BatchNorm at small batch size:** Sensitivity experiments show negligible impact, but instance or group normalization is recommended for future work.
5. **No external validation cohort:** All evaluation is within ADHD-200; generalization to ABIDE or UK Biobank has not been demonstrated.
6. **HiResCAM resolution:** The finest map is at Block 1 (16 × 16 × 14) Sub-voxel precision requires architectural redesign.
7. **Sustainability estimate:** Energy figures are hardware-specific and do not constitute a full lifecycle analysis.

The 14.4 pp LOSO site-to-site spread motivates ComBat harmonization and site-adversarial training as the next engineering priority for closing the telepathology deployment gap. Extension to ASD (9) and comorbid ASD+ADHD classification will leverage the same pipeline on the Autism Brain Imaging Data Exchange (ABIDE) dataset. Integration with EEG biomarkers (35) as a second modality would add millisecond-resolution temporal information complementary to BOLD spatial patterns.

### 4.9 Conclusion

We presented a sustainable, explainable, and telepathology-ready AI pipeline for neurodevelopmental disorder diagnosis from raw voxel-level rs-fMRI, directly addressing all four core themes of this Research Topic: AI advances: 98.7% within-cohort (same-site-contaminated), 78.4% LOSO (primary), 76.1% over 5 well-powered sites; cost-effectiveness (two-stage I/O, 11 × storage reduction, ≈3.7 × energy savings, < 1 s inference); telepathology access in resource-limited settings (LOSO evaluation without specialist preprocessing); and data security (local processing, saliency-only transmission).

The 20.3 pp gap between within-cohort and LOSO performance quantifies the site-correlated signal contamination in the within-cohort evaluation and the scanner harmonization gap that the field must close for sustainable telepathology deployment. HiResCAM voxel-level saliency maps, validated by Dice coefficient analysis across all 231 validation subjects (mean 0.61 ± 0.08, Wilcoxon *p* < 0.001), localize to the right caudate nucleus and DLPFC, and provide the auditable predictions required for clinical digital pathology workflows. These results establish a reproducible baseline and a clear roadmap: dataset harmonization through ComBat (11) and site-adversarial training, not further architectural tuning, is the next critical step toward sustainable AI-driven neuro-digital pathology.

## Data Availability

Publicly available datasets were analyzed in this study. This data can be found here: http://fcon_1000.projects.nitrc.org/indi/adhd200/.
